# A global dataset for crop production under conventional tillage and no tillage systems

**DOI:** 10.1038/s41597-021-00817-x

**Published:** 2021-01-28

**Authors:** Yang Su, Benoit Gabrielle, David Makowski

**Affiliations:** 1 UMR ECOSYS, INRAE AgroParisTech, Université Paris-Saclay, 78850 Thiverval-Grignon, France; 2 UMR Agronomie, INRAE AgroParisTech, Université Paris-Saclay, 78850 Thiverval-Grignon, France; 3 Applied mathematics and computer science (MIA 518), INRAE AgroParisTech, Université Paris-Saclay, 75005 Paris, France

**Keywords:** Agriculture, Climate-change mitigation

## Abstract

No tillage (NT) is often presented as a means to grow crops with positive environmental externalities, such as enhanced carbon sequestration, improved soil quality, reduced soil erosion, and increased biodiversity. However, whether NT systems are as productive as those relying on conventional tillage (CT) is a controversial issue, fraught by a high variability over time and space. Here, we expand existing datasets to include the results of the most recent field experiments, and we produce a global dataset comparing the crop yields obtained under CT and NT systems. In addition to crop yield, our dataset also reports information on crop growing season, management practices, soil characteristics and key climate parameters throughout the experimental year. The final dataset contains 4403 paired yield observations between 1980 and 2017 for eight major staple crops in 50 countries. This dataset can help to gain insight into the main drivers explaining the variability of the productivity of NT and the consequence of its adoption on crop yields.

## Abstract (summary)

**Table:** 

Measurement(s)	yield trait • crop
Technology Type(s)	digital curation
Factor Type(s)	precipitation balance • temperature • crop species • soil texture • management of crop rotation • management of soil cover • management of field fertilization • management of weed and pest control • management of crop irrigation • geographic location
Sample Characteristic - Organism	Viridiplantae
Sample Characteristic - Environment	cultivated environment
Sample Characteristic - Location	global

Machine-accessible metadata file describing the reported data: 10.6084/m9.figshare.13303118

## Background & Summary

Often featured among promising climate change mitigation measures, NT systems, including conservation agriculture (CA), contribute to environmental preservation and sustainable agricultural production^1,2^. NT is expected to mitigate soil degradation, improve soil structure and water retention properties^3–5^. Several studies indicate that this cropping system can provide a large range of positive environmental externalities such as increased biodiversity, enhanced carbon sequestration and improved soil quality through an increase in soil organic matter^6–10^. However, the productivity of NT systems compared to conventional cropping systems is still controversial. Since the productivity of NT depends on several interacting factors such as climatic conditions^11^, soil characteristics^1,12^, and other agricultural management activities^13–19^, the potential of NT to increase agricultural productivity remains highly uncertain.

Several studies^1,12,20–22^ have been conducted to synthetize the current evidence on the productivity in NT systems. Some of these studies relied on global datasets including results of field experiments comparing NT and CT cropping systems over a large range of soil and climate conditions. However, these datasets do not include the most recent published experiments, and provide no or limited information on soil characteristics, climate variables, and management practices. In particular, information on fertilizer inputs, weed and pest control, and intra- and inter- annual climatic variability are frequently missing. Other studies comparing NT and CT rely on a limited number of experiments, are only conducted at a regional scale, or did not make their data fully available^23–25^. Thus, a global dataset reporting findings from the most recent field experiments and including information about a wide range of climatic parameters, soil characteristics and agricultural management practices is still lacking.

To address this gap, we present an updated and extended dataset comparing CT and NT productivity including the most recently published experimental studies, and a detailed description of their environmental characteristics and management practices. Our dataset contains 4403 paired (NT vs. CT) yield observations collected between 1980 and 2017 for eight major staple crops in 50 countries. For each experiment, we provide information on soil texture, pH, the year and month of crop planting and harvesting, the location of the experiment, fertilization, weed and pest control practices, crop type, crop rotation, crop residue management, and crop irrigation. Besides soil characteristics and information on management practices, we also report a large range of climate variables derived from several external databases. These include precipitation, potential evapotranspiration, average temperature, maximum temperature, and minimum temperature during the crop growing season. This dataset can prove useful to disentangle the effects of soil, climate and agronomic drivers of crop yields when comparing NT with CT systems.

## Methods

### Data collection

**Fig. 1:**
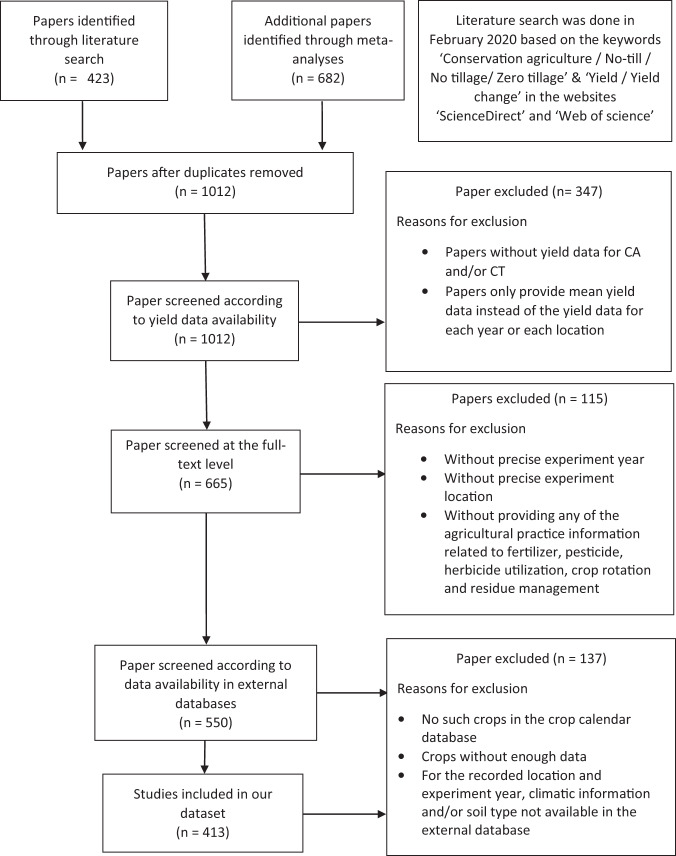
Flow chart of paper collection and selection.

**Fig. 2:**
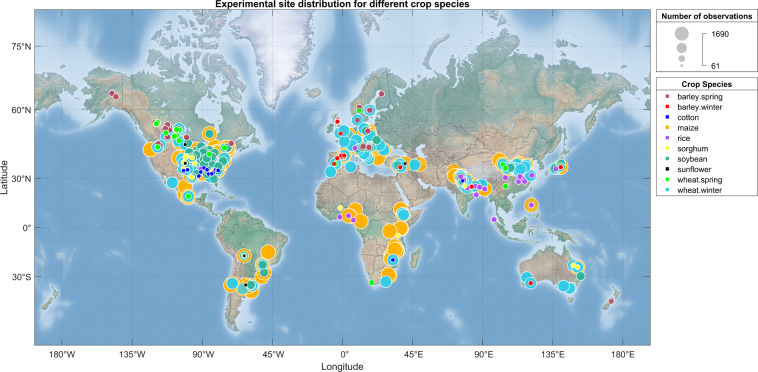
Distribution of experimental sites. The size of the circle indicates the number of observations, and the colours indicates the crop species.

The literature search was done in February 2020 using the following keywords ‘Conservation agriculture/No-till/No tillage/Zero tillage’ & ‘Yield/Yield change’ in the websites ‘ScienceDirect’, ‘Science Citation Index (web of science)’. A total of 1012 potentially relevant papers were identified by reviewing the title and abstract, and these papers were then screened according to the procedure summarized in Fig. 1. Papers not reporting yield data for CT and NT systems were excluded, as well as papers reporting experiments on reduced tillage (RT) systems. Papers reporting only mean yield data across different years or sites were also excluded. We then checked whether information on fertilization, weed and pest control, crop irrigation, crop rotation and crop residue management were reported for both CT and NT practices. After these screening and selection steps, all relevant data were manually extracted from the selected papers, including general information about the paper, location and year of the experiment, the number of years under NT when the crop was sown, soil characteristics, crop growing season, crop type, crop management practices and crop yield of CT and NT. However, due to a large number of missing data, the crop growing season, climatic variables and soil characteristics were finally collected through several external databases (Online-only Table 1). The growing season information was generated from a crop calendar database^26,27^ based on the crop type and the locations of the experiments reported in the papers. The precipitation, average temperature in the growing season were extracted from the UDel_AirT_Precip data provided by NOAA/OAR/ESRL PSL^28^. The maximum and minimum air temperature during the growing season were generated from CPC Global Temperature data provided by NOAA/OAR/ESRL PSL^29^ and the potential evapotranspiration data over the growing season were extracted from GLEAM database^30,31^. Soil textures were collected from the HWSD database^32^ using the locations of the experimental sites reported in the selected papers (see Online-only Table 1 for additional details). The experiments for which it was not possible to obtain the requested information from the external databases were excluded. The final dataset includes the results extracted from 413 papers (published between 1983 to 2020), 4403 paired yield observations from NT and CT for 8 major crop species, including 370 observations for barley (232 observations for spring barley and 138 for winter barley), 94 observations for cotton, 1690 observations for maize, 195 observation for rice, 160 observations for sorghum, 583 observations for soybean, 61 observations for sunflower, 1250 observations for wheat (1041 observations for winter wheat and 209 observations for spring wheat) in 50 countries from 1980 to 2017 (Fig. 2).

### Data records

All data are available on the figshare repository^33^, which can be accessed through the link: 10.6084/m9.figshare.12155553. Four files are provided:

1. “Database.csv” includes the data.
2. “Summary of the database.docx”, includes the summary of dataset which explains the definition or assumption for each column in the dataset.
3. “List of references.docx” reports the references of the studies from which data were extracted.
4. “Code.zip”, includes all the codes used in this study.

Online-only Table 1 shows the metadata of our dataset. Six main categories of data are provided:

Category I covers authors, publishing journal and the publishing year.

Category II reports general information about the experiments, including country, location (villages or cities), latitude, longitude of experiment site, soil type and pH at experimental sites, number of replicates, crop types, the initial year of NT practice, crop planting/harvesting month/year, and the period since the initial year of NT practice.

**Fig. 3:**
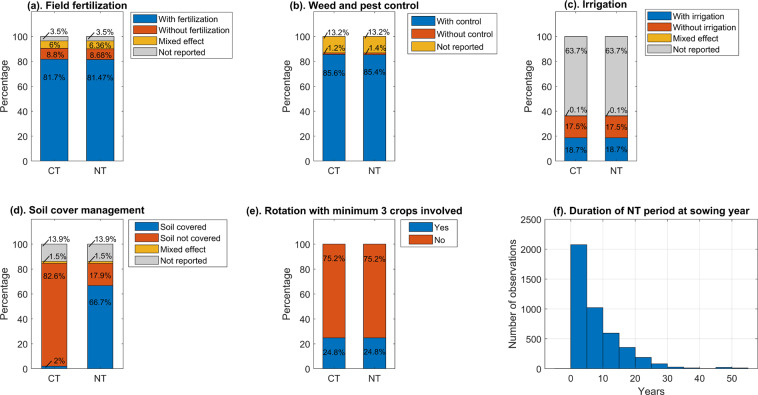
Data availability and break-down of the different crop management practices and NT implementation periods reported in the dataset.

Category III covers information about agricultural management activities under both NT and CT systems (data availabilities of those activities were shown in Fig. 3):

- Crop rotation with at least 3 crops involved (based on the crop rotation principle of CA defined by FAO^34^): “Yes”, “No”, “Not reported”. The details of crop sequence are also provided.

- Soil cover: “Yes”, “No”, “Mixed”, “Not reported”. Details of residue management for the previous crops are also provided.
- Weed and pest control: “Yes”, “No”, “Mixed”, “Not reported”.
- Field fertilization: “Yes”, “No”, “Mixed”, “Not reported”. The details of N input and other fertilizer input are also provided.
- Crop irrigation: “Yes”, “No”, “Mixed”, “Not reported”. The details of the amount of water applied are also provided.

Category IV contains detailed information about the experiment site, experiment setting, management activities, depending on the papers, it may also include the type and quantity used of fertilizer, herbicide, or pesticide.

Category V corresponds to data related to crop yield. It includes the paired crop yields under CT (*Yield*_*CT*_) and NT (*Yield*_*NT*_) systems. The relative yield changes is defined as $\frac{Yiel{d}_{NT}\,-\,Yiel{d}_{CT}}{Yiel{d}_{CT}}$. The column “Yield increase with NT” reports whether the differences between *Yield*_*CT*_ and *Yield*_*NT*_ are positive or negative (“Yes” indicates that crop yield is increased with NT practice, while “No” indicates that yield is not increased).

Category VI includes data extracted from the external databases, including crop growing season, climate variables (including precipitation, potential evapotranspiration, minimum/average/maximum temperature) during the growing season, and soil texture.

Crop growing season is defined by a start month and end month, which were extracted from the external crop calendar databases^26^ of spring barley, winter barley, cotton, maize, rice, sorghum, soybean, sunflower, spring wheat and winter wheat based on the crop type and study sites. Data on the crop calendar corresponds to averaged data and does not change intra-annually, thus the growing season extracted may be different from the actual growing season.

The climatic variables from the external databases are:

- Accumulated precipitation (P) during the growing season (sum of monthly precipitations during the growing season),
- Accumulated potential evapotranspiration (E) (sum of monthly evapotranspiration rates during the growing season),
- Precipitation balance (PB), defined as PB = P – E^12^,
- Average air temperature (Tave) during the growing season,
- Maximum air temperature (Tmax): the maximum value among the daily temperatures in the growing season,
- Minimum air temperature (Tmin): the minimum value among the daily temperatures in the growing season.

Soil texture was extracted from an external database based^32^ on the experiments’ locations. In total seven texture classes were included: sandy loam, loam, silt loam, sandy clay loam, clay loam, sandy clay and clay.

## Technical Validation

To ensure the reliability of the information collected from the papers, we carefully checked and compared all the collected data with the original paper several times. Quality control of the database was conducted based on outlier detection. For each crop, the outliers of crop yield in CT system and NT system were identified based on the Interquartile Rule^35^ outlier detection method. For each crop species, an interquartile range (IQR) is defined as the difference between the first and third quartile of crop yield, and a threshold is calculated by adding 1.5 IQR to the third quartile. Any yield data beyond this threshold is flagged as an outlier for the crop species considered. The ratio of crop yield in NT and CT systems $\left(\frac{Yiel{d}_{NT}}{Yiel{d}_{CT}}\right)$ were also calculated. All outliers and all the observations with a ratio higher than 2 were checked and compared with the values reported in the original papers one more time.

**Fig. 4:**
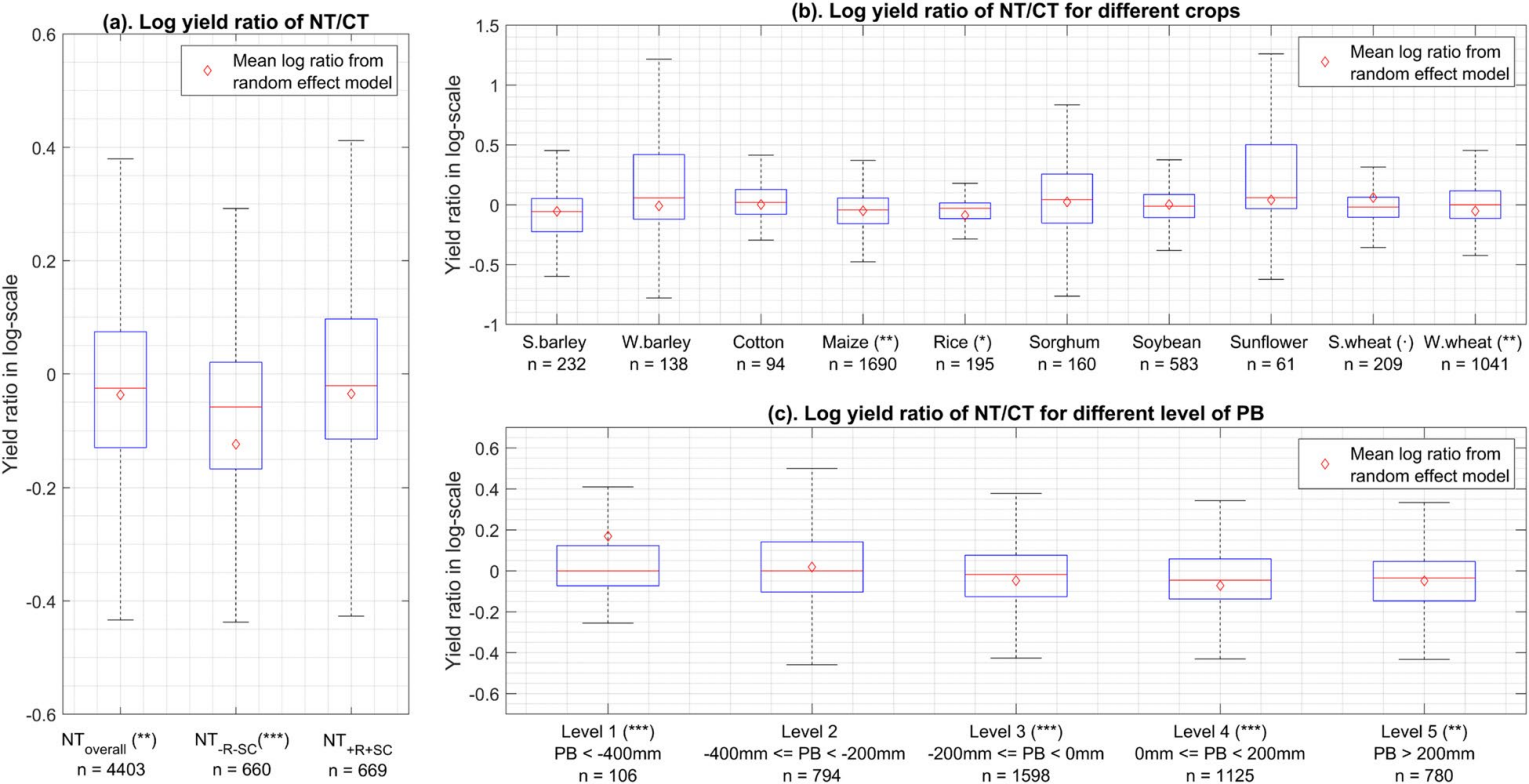
Comparison of crop yield between NT and CT systems. The boxplots indicate the distributions (min, 1^st^ quartile, median, 3^rd^ quartile, max) of the log yield ratio of NT to CT. The mean log yield ratios of NT to CT were calculated based on a linear mixed effect model and marked as the red diamonds in the boxplots. Statistical tests were conducted to test the significance of the estimated values, ***indicates P-value < 0.001, **indicates P-value < 0.01, *indicates P-value < 0.05, indicates P-value < 0.1. Plot (**a**) shows the mean log ratios for different types of NT systems vs. CT systems. *NT*_*overall*_ represents all the experiments involving NT systems in the dataset, *NT*_*-R-SC*_ represents the NT systems without crop rotation and without soil cover, *NT*_+*R*+*SC*_ represents the CA systems or NT systems with both crop rotation and soil cover, and CT is the corresponding control in the experiments. Plot (**b**) shows the mean log ratios for different crop species. S. indicates spring, while W. indicates winter. Plot (**c**) shows the mean log ratios for different levels of PBs, corresponding to different level of water stress.

The crop yield values reported in our dataset are consistent with results of previous published studies. Comparing crop yield data of NT and CT, the adoption of NT practice overall leads to a yield decrease (Fig. 4a). A similar trend of crop production decrease with NT was reported in previous studies^1,12,20,36^. We also find that the combination of NT with crop rotation and soil cover (known as CA) trends to increase crop yield compared to NT practice without rotation and soil cover (Fig. 4a), which is also in line with previous studies^1,16,37^. Further analysis conducted on each crop confirms that NT tends to decrease the yield of maize^1^, rice^21^, and wheat^1^ (Fig. 4b). The productivity of NT is found higher under dry conditions compared with wetter conditions (Fig. 4c), and similar trends were reported in previous studies^1,12^.

### Usage notes

Our dataset can be used to analyze the factors influencing the productivity of NT (or CA) vs. CT. It is possible to train machine learning models to predict the probability of yield increase with NT (or CA) system (e.g. Supplementary Figs. 1 and 2) or the range of yield changes resulting based on the soil type, climate and agronomic inputs provided by this dataset. Global maps of probability of yield increase with NT (or CA) or the range of yield changes can be generated based on the outputs of machine learning models trained with our dataset, and enable policy-makers or agricultural advisors to identify the most promising regions for CA implementation. Details of how to train machine leaning models with our dataset are provided in Supplementary Materials^38–450^.

The crop yield data for 2018 and later can be extracted from the identified papers, but since some key climatic variables are missing in the external database for this time period (in particular, evapotranspiration), those data are not listed in the dataset provided. We will update the dataset once we have the latest data access to the missing climate variables.

Importantly, our dataset could be easily updated using data produced by new experiments. We welcome anyone interested to share data or papers not included in this meta-database to send them to the corresponding author (YS, yang.su@inrae.fr). We will maintain and add the new observations in the future to expand our dataset with the latest experimental data.

## Supplementary information

Supplementary Materials

## Data Availability

Scripts using the R and MATLAB programming language are provided to produce figures and extract data from external databases. Additional code and related files are available from the corresponding author upon request.

## Online-only Table

**Online-only Table 1:** Metadata of the dataset.

Category	Column	Data collected	Units	Notes	Source/Formula
I. General information of the paper	A	Author			Paper
	B	Journal			
	C	Publish year			
II. Experiment information	D	Country of experimental site			
	E	Location of experimental site			
	F	Latitude of experimental site	Degree		
	G	Longitude of experimental site	Degree		
	H	Soil type or texture			
	I	Surface pH of experimental site			
	J	Number of experiment replications			
	K	Crop type		Including 8 crops: barley (spring & winter barley), cotton, maize, rice, sorghum, soybean, sunflower, wheat (spring & winter wheat).	
	L	Initial year of NT practice	Year	If not mentioned in the paper, then this is assumed to be the initial year of the experiment.	
	M	Crop sowing year	Year		
	N	Crop harvesting year	Year		
	O	Years since NT started	Year		*Year*_*sowing*_–*Year*_*NT,initial*_+1
	P	Crop planting month and harvesting month	Month	Crop growing season reported in the paper.	Paper
III. Information about agricultural management activities	Q	Crop rotation with at least 3 crops involved in CT practice		Here we set crop rotation as “Yes” only when the crop species (including the cover crops) involved in the crop rotation sequence is more than 2 species in accordance with the FAO’s definition of Conservation Agriculture. More details about the crop sequence can be found in column S.	
	R	Crop rotation with at least 3 crops involved in NT practice		Two categories: “Yes”, “No”.	
	S	Details of crop rotation sequence			
	T	Cover crop before sowing		Set to “Yes” if a cover crop is present in the experiment	
	U	Soil cover in CT practice		Four categories: “Yes”, “No”, “Mixed”, “Not reported”. Here we set soil cover as “Yes” when more than 30% of the soil is covered even after tillage, or when plastic/residue mulch exists. We set soil cover as “Mixed” for yield data corresponding to the average yields of both categories “Yes” and “No”.	
	V	Soil cover in NT practice		Four categories: “Yes”, “No”, “Mixed”, “Not reported”. Here we set soil cover as “Yes” only when more than 30% of the soil is covered in current cropping season and residues from current cropping season are retained. We set soil cover as “Mixed” for yield data corresponding to the average yields of both categories “Yes” and “No”.	
	W	Details of residue management of previous crop in CT practice			
	X	Details of residue management of previous crop in NT practice			
	Y	Weed and pest control in CT practice		Three categories: “Yes”, “No”, “Not reported”.	
	Z	Weed and pest control in NT practice		Three categories: “Yes”, “No”, “Not reported”.	
	AA	Details of weed and pest control in CT practice			
	AB	Details of weed and pest control in NT practice			
	AC	Field fertilization in CT practice		Four categories: “Yes”, “No”, “Mixed”, “Not reported”. We set field fertilization as “Mixed” for yield data corresponding to the average yields of both categories “Yes” and “No”.	
	AD	Field fertilization in NT practice		Four categories: “Yes”, “No”, “Mixed”, “Not reported”. We set field fertilization as “Mixed” for yield data corresponding to the average yields of both categories “Yes” and “No”.	
	AE	N input		Four categories: “Yes”, “No”, “Mixed”, “Not reported”. We set N input as “Mixed” for yield data corresponding to the average yields of both categories “Yes” and “No”.	
	AF	N input in details with the unit kg N ha-1			
	AG	Details about field fertilization			
	AH	Crop irrigation in CT practice		Four categories: “Yes”, “No”, “Mixed”, “Not reported”. We set crop rotation as “Mixed” for yield data corresponding to the average yields of both categories “Yes” and “No”.	
	AI	Crop irrigation in NT practice		Four categories: “Yes”, “No”, “Mixed”, “Not reported”. We set crop rotation as “Mixed” for yield data corresponding to the average yields of both categories “Yes” and “No”.	
	AJ	Details of water applied in CT practice		Water applied in the field with the unit mm/ha/year	
	AK	Details of water applied in CT practice		Water applied in the field with the unit mm/ha/year	
IV. Other detailed information	AL	More detailed information about the experiment setting and the agricultural activities			
V. Information about crop yield	AM	Crop yield under CT practice	kg/ha		
	AN	Crop yield under NT practice	kg/ha		
	AO	Relative yield change			..
	AP	Yield increase with NT practice?		Yes = yield increased with NT No = yield not increased with NT	
	AQ	Outlier of crop yield in CT practice		Set to “Yes” if it is an outlier for corresponding crop species	
	AR	Outlier of crop yield in NT practice		Set to “Yes” if it is an outlier for corresponding crop species	
VI. Information from external databases: climatic variables and soil texture	AS	Crop growing season start month	Month	Crop growing season was extracted from the external crop calendar databases of spring barley, winter barley, cotton, maize, rice, sorghum, soybean, sunflower, spring wheat and winter wheat based on the crop type and location	University of Wisconsin-Madison^26,27^
	AT	Crop growing season end month	Month	Crop growing season were extracted from the external crop calendar databases of spring barley, cotton, maize, rice, sorghum, soybean and winter wheat based on the crop type and location	University of Wisconsin-Madison^26,27^
	AU	Precipitation over the growing season	mm	Precipitation was extracted from the external database based on the location, year of the experiment and crop growing season from the external database. The precipitations in each month were added together.	NOAA/OAR/ESRL PSL^28^
	AV	Potential evapotranspiration over the growing season	mm	Potential evapotranspiration was extracted from the external database based on the location, year of the experiment and crop growing season from the external database. The potential evapotranspiration rates in each month were added together.	GHENT university/ESA GLEAM^30,31^
	AW	Precipitation balance	mm	Indicated the amount of available water in the growing season for rainfed field	*P−E*
	AX	Average air temperature during the growing season	°C	Average temperature was extracted from the external database based on the location, year of the experiment and crop growing season from the external database. The temperature in each month were averaged.	NOAA/OAR/ESRL PSL^28^
	AY	Maximum air temperature during the growing season	°C	Maximum temperature was extracted from the external database based on the location, year of the experiment and crop growing season from the external database. The temperatures in each month of the growing season were compared, the maximum one was recorded.	NOAA/OAR/ESRL PSL^29^
	AZ	Minimum air temperature during the growing season	°C	Minimum temperature was extracted from the external database based on the location, year of the experiment and crop growing season from the external database. The temperatures in each month of the growing season were compared, the minimum one was recorded.	NOAA/OAR/ESRL PSL^29^
	BA	Soil texture		Soil texture was extracted from the external database based on the location of the experiment from the external database. Categories included in this database: Sandy Loam; Loam; Silt Loam; Sandy Clay Loam; Clay Loam; Sandy Clay; Clay	The University of Tokyo^32^
