# Supplementary material for: A global dataset for crop production under conventional tillage and no tillage systems
Source: Sci Data. 2021 Jan 28;8:33. doi: 10.1038/s41597-021-00817-x (PMC7844240; doi:10.1038/s41597-021-00817-x)
Supplement: Supplementary file 1 — Supplementary Materials [file 41597_2021_817_MOESM1_ESM.pdf]

A global dataset for crop production under conventional tillage and no tillage practice

Authors

Yang Su[a], Benoit Gabrielle[a], David Makowski[b,c]

Affiliations

[a] UMR ECOSYS, INRAE AgroParisTech, Université Paris-Saclay, 78850 Thiverval-Grignon, France

[b] UMR Agronomie, INRAE AgroParisTech, Université Paris-Saclay, 78850 Thiverval-Grignon, France

[c] Applied mathematics and computer science (MIA 518), INRAE AgroParisTech, Université Paris-Saclay, 75005 Paris, France.

Corresponding Author: Yang Su (yang.su@inrae.fr)

Supplementary figure captions

Supplementary figure 1 Probability of yield increase of winter barley under NT vs. CT practice in the average climate conditions of 1981-2010.

Supplementary figure 2 Probability of yield increase of winter barley under CA vs. CT practice in the average climate conditions of 1981-2010.

Supplementary figure 1

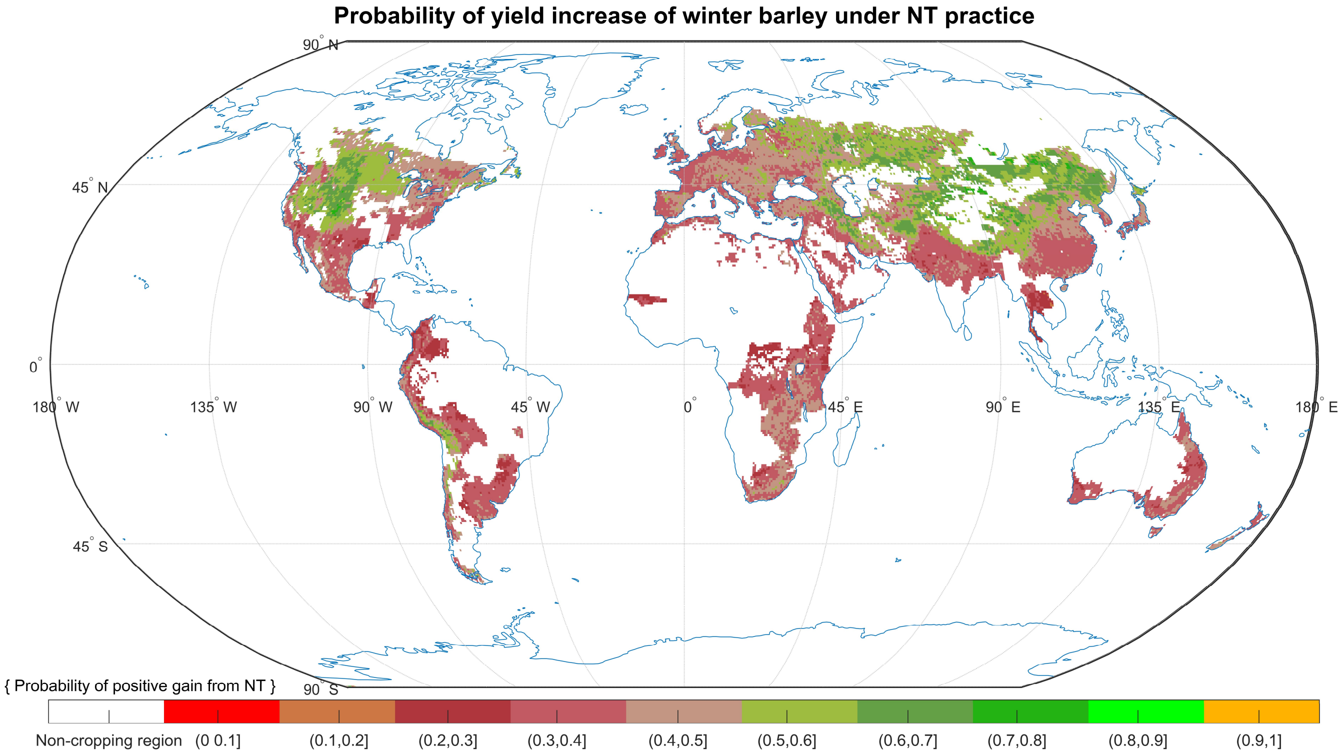

Supplementary figure 2

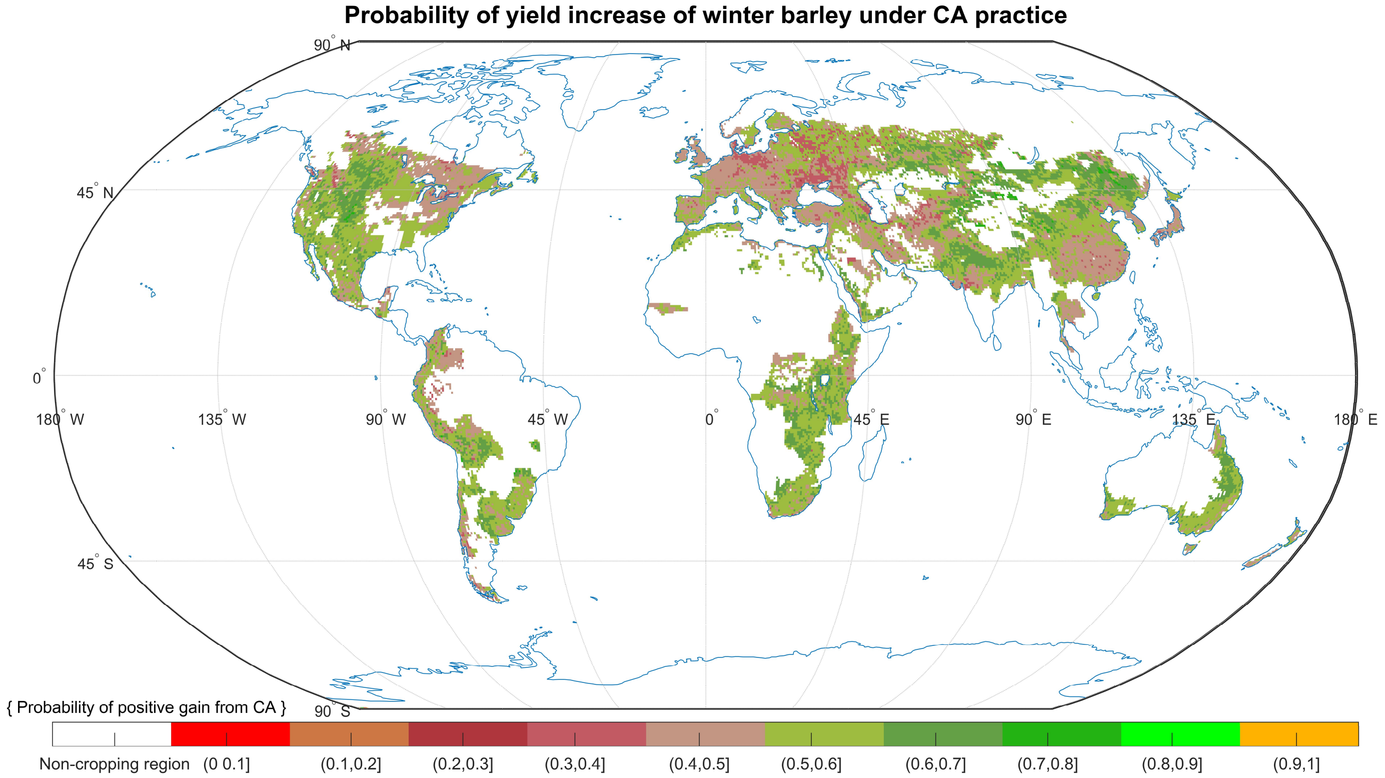

Method to train machine learning models based on our dataset

The machine learning algorithms, e.g. random forest, can be trained based on the 4403 paired crop yield observations collected from papers [38-450]. It can be used to analyze the yield ratios of NT vs. CT as the function of climatic variables, crop types, soil textures, and agricultural management activities. And this can be done in R software with the package "randomForest". To train the model, set the climatic variables during the growing season such as PB, Tmin/Tave/Tmax as numerical predictor variables; Set crop type, soil texture, and agricultural management activities including crop irrigation, field fertilization, control of pests and weeds, crop rotation and soil cover as categorical predictor variables; Set the column AP (Yield increase with NT) as response variable. When training, each tree in a random forest learns from a random sample of the data points, the samples are drawn with replacement (bootstrapping), only a subset of all the inputs are considered for splitting each node in each decision tree. Predictions are made by averaging the predictions of all decision trees.

The sample code for training the random forest model is available in "Code.zip" in figshare repository provided in the main manuscript. When setting the "proximity" as Ture, the model output is the probability of yield gain from NT vs. CT.

Method to producing the global maps of NT (or CA) vs. CT practices on our dataset

After the training the random forest model, supply the trained model the global climate variables and soil texture, plus the self-defined agricultural management activities, then the model can be used to predicting the performance of NT (or CA, when set with crop rotation and soil cover) vs. CT at the global scale under different agriculture management activities.

The sample codes for using the random forest model to predict (in R software) and map (MATLAB) the global performance of NT (or CA) vs. CT are available in "Code.zip" in figshare repository provided in the main manuscript. More codes can be requested by sending email to corresponding author.
